# The synthetic aminoglycoside ELX-02 induces readthrough of G550X-CFTR producing superfunctional protein that can be further enhanced by CFTR modulators

**DOI:** 10.1152/ajplung.00038.2023

**Published:** 2023-04-04

**Authors:** Jianguo Chen, Kari Thrasher, Lianwu Fu, Wei Wang, Soheil Aghamohammadzadeh, Hui Wen, Liping Tang, Kim M. Keeling, Emily Falk Libby, David M. Bedwell, Steven M. Rowe

**Affiliations:** ^1^Department of Medicine, University of Alabama at Birmingham, Birmingham, Alabama, United States; ^2^Department of Biochemistry and Molecular Genetics, University of Alabama at Birmingham, Birmingham, Alabama, United States; ^3^Gregory Fleming James Cystic Fibrosis Research Center, University of Alabama at Birmingham, Birmingham, Alabama, United States; ^4^Eloxx Pharmaceuticals, Inc., Watertown, Massachusetts, United States

**Keywords:** aminoglycosides, CFTR, nonsense mutations, premature termination codons, translational readthrough

## Abstract

Ten percent of cystic fibrosis (CF) patients carry a premature termination codon (PTC); no mutation-specific therapies exist for these individuals. ELX-02, a synthetic aminoglycoside, suppresses translation termination at PTCs (i.e., readthrough) by promoting the insertion of an amino acid at the PTC and restoring expression of full-length CFTR protein. The identity of amino acids inserted at PTCs affects the processing and function of the resulting full-length CFTR protein. We examined readthrough of the rare G550X-CFTR nonsense mutation due to its unique properties. We found that forskolin-induced swelling in G550X patient-derived intestinal organoids (PDOs) was significantly higher than in G542X PDOs (both UGA PTCs) with ELX-02 treatment, indicating greater CFTR function from the G550X allele. Using mass spectrometry, we identified tryptophan as the sole amino acid inserted in the G550X position during ELX-02- or G418-mediated readthrough, which differs from the three amino acids (cysteine, arginine, and tryptophan) inserted in the G542X position after treatment with G418. Compared with wild-type CFTR, Fischer rat thyroid (FRT) cells expressing the G550W-CFTR variant protein exhibited significantly increased forskolin-activated Cl^−^ conductance, and G550W-CFTR channels showed increased PKA sensitivity and open probability. After treatment with ELX-02 and CFTR correctors, CFTR function rescued from the G550X allele in FRTs reached 20–40% of the wild-type level. These results suggest that readthrough of G550X produces greater CFTR function because of gain-of-function properties of the CFTR readthrough product that stem from its location in the signature LSGGQ motif found in ATP-binding cassette (ABC) transporters. G550X may be a particularly sensitive target for translational readthrough therapy.

**NEW & NOTEWORTHY** We found that forskolin-induced swelling in G550X-CFTR patient-derived intestinal organoids (PDOs) was significantly higher than in G542X-CFTR PDOs after treatment with ELX-02. Tryptophan (W) was the sole amino acid inserted in the G550X position after readthrough. Resulting G550W-CFTR protein exhibited supernormal CFTR activity, PKA sensitivity, and open probability. These results show that aminoglycoside-induced readthrough of G550X produces greater CFTR function because of the gain-of-function properties of the CFTR readthrough product.

## Graphical abstract

**Figure:**
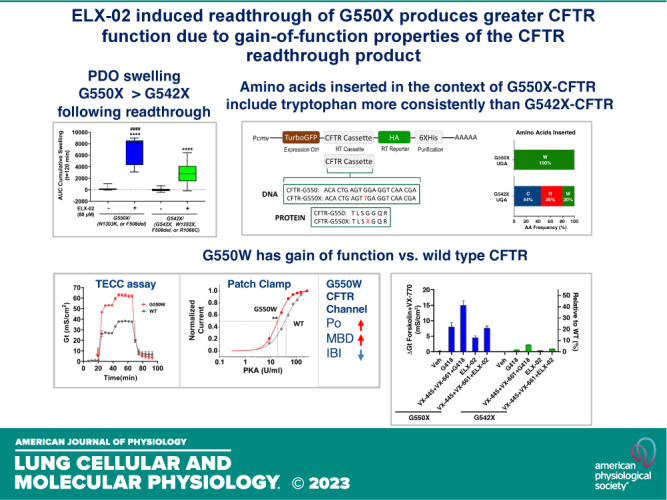

## INTRODUCTION

Cystic fibrosis (CF) is a life-limiting hereditary disease caused by loss-of-function mutations in the cystic fibrosis transmembrane conductance regulator (CFTR) gene. The CFTR protein is an epithelial chloride channel from the ATP-binding cassette (ABC) transporter family. CFTR is composed of two membrane-spanning domains [transmembrane domain (TMD)1 and TMD2] that form the chloride channel pore, two nucleotide-binding domains (NBD1 and NBD2) that bind and hydrolyze ATP, and a regulatory domain (R) with several protein kinase A (PKA) phosphorylation sites (1, 2). Over 2,000 CFTR variants, including 401 confirmed CF-causing mutations, have been reported [The Clinical and Functional Translation of CFTR (CFTR2), April 29, 2022; available at https://cftr2.org]. Among point mutations, nonsense mutations are common. A nonsense mutation creates an in-frame premature termination codon (PTC) that both terminates translation of CFTR mRNA before the full-length protein is generated and reduces PTC-containing CFTR mRNA abundance by triggering nonsense-mediated mRNA decay (NMD). Although the development of CFTR modulator drugs has provided a treatment option for ∼90% of CF patients, no approved mutation-specific therapies exist for the 10% of people with CF who carry a nonsense mutation. Translation termination at PTCs can be suppressed in mammalian cells by aminoglycosides (3–5). Aminoglycosides bind to the mammalian ribosome and reduce translational fidelity, resulting in acceptance of a near-cognate aminoacyl tRNA at the site of the PTC, a process known as “readthrough” (RT). Readthrough restores in-frame translation elongation downstream of a PTC, allowing a full-length protein to be generated.

The identity of the amino acids inserted at PTCs, which can affect the processing and function of the resulting full-length CFTR protein, is largely context dependent (6, 7). Previously we reported that the aminoglycoside G418 induces the insertion of three different amino acids [Cys (C), Arg (R), and Trp (W)] in different proportions at the G542 position in the context of a G542X-CFTR PTC (UGA codon); these CFTR variants (G542C, G542R, and G542W) exhibited reduced CFTR function by varying extents compared with wild-type (WT)-CFTR (7, 8). G418 and other aminoglycosides such as gentamicin have not been translated into a useful clinical readthrough therapy because of off-target effects that lead to renal and cochlear toxicity in humans (9). ELX-02 (NB-124) is a novel aminoglycoside derivative that displays preferential binding to the eukaryotic ribosome (10), reducing toxicity and improving readthrough compared with conventional aminoglycosides (11).

Previously we reported that ELX-02 can restore CFTR expression in human bronchial epithelial (HBE) cells (12), Fischer rat thyroid (FRT) cells (12), and transgenic mice (12) carrying the G542X nonsense mutation, the most common CF nonsense allele. ELX-02 also enhanced CFTR-dependent organoid swelling in G542X patient-derived intestinal organoids (13). Furthermore, partial CFTR function could be restored in R1162X, W679X, or G542X patient-derived intestinal organoids, as well as in patient-derived intestinal organoids expressing W1282X-CFTR when combined with the NMD inhibitor SMG1i and CFTR modulators (VX-661/VX-445/VX-770) (14). ELX-02 is being developed as a therapy for genetic diseases caused by nonsense mutations such as cystic fibrosis (CF) and nephropathic cystinosis (15). A recent phase 2 clinical trial evaluating the safety and biological activity of ELX-02 alone or in combination with ivacaftor in CF subjects with a G542X mutation on one or both alleles indicated that ELX-02 was generally well tolerated, with no serious treatment-related adverse events noted. ELX-02 monotherapy study met a key secondary end point by showing a statistically significant reduction in mean sweat chloride concentration (SCC), but the ELX-02 + ivacaftor combination therapy study did not achieve statistical significance for efficacy end points including changes from baseline in SCC or forced expiratory volume in 1 s (FEV_1_). Investigators thought that the efficacy failure could be caused by very low ELX-02 exposure in the lung because this trial found that the steady-state lung drug levels in patients from this trial were on average 20%, or 2 μM, of the lowest levels at which drug activity has previously been seen in preclinical testing [Eloxx Pharmaceuticals Reports Topline Results from Phase 2 Combination Clinical Trial of ELX-02 in Class 1 Cystic Fibrosis (CF) Patients; source: Eloxx Pharmaceuticals, Inc., September 22, 2022]. Investigation for improving ELX-02 exposure in the lung, particularly increasing ELX-02 level in respiratory tract epithelial cells, is needed.

Compared with G542X, the G550X allele is a rare CFTR nonsense allele. Relative allele frequencies in worldwide incidence are 0.1% for G550X (*n* = 6) and 36% for G542X (*n* = 3,610) in the PTC variants (*n* = 9,964) reported in The Clinical and Functional Translation of CFTR (CFTR2) (April 29, 2022; available at https://cftr2.org). Recently, we discovered that G550X patient-derived intestinal organoids are highly responsive to forskolin-induced swelling when treated with ELX-02 compared with the response seen in G542X organoids. However, it is unclear why the G550X allele is so uniquely responsive. A recent report demonstrated that ELX-02 significantly enhanced CFTR mRNA abundance and protein function of S1196X and S466X CFTR variants in human nasal epithelial cells and that the U UGA C sequence provides a favorable context for ELX-02-induced CFTR readthrough (16). The G550 residue of CFTR is located in the signature LSGGQ sequence just distal to the Walker B motif in the first nucleotide-binding domain (NBD1), which contributes to both CFTR channel activation and function in WT-CFTR. We therefore hypothesized that readthrough at the G550X PTC and introduction of a near-cognate amino acid substitution produces a CFTR protein variant with similar or even increased activity relative to WT-CFTR. Here, we have identified the amino acids inserted during ELX-02-induced G550X PTC suppression, evaluated CFTR variant function, and assessed whether the addition of clinically approved CFTR correctors can further augment readthrough and function of G550X-CFTR. These data provide a guide for how readthrough analysis of unique CFTR nonsense mutations can be addressed in a systematic approach.

## MATERIALS AND METHODS

### Patient-Derived Organoid Forskolin-Induced Swelling Assay

All intestinal patient-derived organoid (PDO) cultures used in this study belong to the Hubrecht Organoid Technology (HUB) CF organoid biobank. Written informed consent for tissue collection, generation, storage, and use of the PDOs was obtained from patients at Wilhelmina Children's Hospital (WKZ)-University Medical Center Utrecht (UMCU). All assays using human PDOs described here were approved by the UMCU ethical committee (TcBio no. 14-008). All PDO cultures were matched with their original tissue by single-nucleotide polymorphism fingerprinting analysis. The forskolin-induced swelling (FIS) assay for PDOs was performed as described previously (13, 17). Briefly, ELX-02 (provided by Eloxx Pharmaceuticals, Inc.) was added at the time of organoid seeding (20–60 organoids per well) in a 96-well assay plate. Organoids were then incubated with ELX-02 for 48 h, followed by a 30-min incubation with calcein green (10 μM). After calcein treatment, forskolin was added at the indicated concentrations and organoid size was directly measured by fluorescence microscopy for up to 2 h. Images were captured at 10-min intervals with a Cell Voyager 7000S (Yokogawa) microscope or a PerkinElmer Operetta CLS High-Content Analysis system. Images were automatically analyzed with Fiji (Fiji Life-Line version, November 25, 2014), an open-source image processing package based on ImageJ. Individual PDOs were detected and tracked for change in cross-sectional area over time (relative to *t* = 0). The average change in PDO size was tracked and represented as a percent change from baseline (100%), from which the area under the curve (AUC) was calculated with GraphPad Prism software (version 9.0).

### Identification and Characterization of the Protein Produced upon ELX-02-Mediated Readthrough of the G550X-CFTR Mutation

A reporter construct containing G550X context (TurboGFP-G550X-HA-6XHis) in HEK293 cells was used to identify the amino acids that are inserted at the PTC of G550X by tandem mass spectrometry analysis.

For creation of TurboGFP-CFTR-HA-6Xhis-expressing HEK293 cells, a CFTR cassette expressing seven codons of G550X-CFTR (3 codons upstream and downstream of the CFTR G550 position) was inserted into a TurboGFP-HA-6XHis construct downstream of TurboGFP. This construct was then ligated into the mammalian expression vector pcDNA3.1zeo(+) (Thermo Scientific, V86020). This plasmid was transfected into HEK293 cells (ATCC, CRL-4573), and zeocin (200 µg/mL) was used to select for stable transformants.

For protein purification, cells stably expressing TurboGFP-G550X-HA-6XHis were treated with ELX-02 at 600 µg/mL for 48 h. Cells were harvested and lysed with M-PER Mammalian Protein Extraction Reagent (Thermo Scientific, 78501) with protease inhibitors (Roche, 11873580001). Cell lysates were clarified and purified with a HiTrap TALON Crude column (Cytiva, 28953766) on an AKTA protein purification system (Cytiva). Elution fractions were then combined and concentrated with Amicon Ultra-0.5 Centrifugal Filter Units (MilliporeSigma, UFC501024).

For LC-MS/MS sample preparation, the concentrated elution was separated via SDS-PAGE and stained with Bio-Safe Coomassie (Bio-Rad, 1610786). The band of interest was excised and destained. The in-gel proteins were then reduced with dithiothreitol (10 mM), alkylated with iodoacetamide (50 mM), and digested with trypsin (Promega, V5280) (12.5 ng/µL). Peptides were then eluted from the gel with 5% formic acid and dried down.

For tandem mass spectrometry, dried peptides were reconstituted in 0.1% formic acid and placed on an LC autosampler. A nanospray column packed with C18 resin was used in conjunction with an LTQ Orbitrap Velos Pro mass spectrometer (Thermo Scientific). The mass spectrometer was set to switch between a full scan [400–1,200 mass-to-charge ratio (*m/z*)] followed by successive MS/MS (200–1,200 *m/z*) scans of the 10 most abundant precursor ions. Mass spectrometry data were analyzed by using Mascot Daemon (Matrix Science) to identify the amino acid inserted at the PTC position.

### Fischer Rat Thyroid Cell Line Development and Treatment

FRT cells expressing WT- or G550-CFTR variants (G550X, G550W) were generated based on a Flp-In system (ThermoFisher) to allow accurate comparison between mutation groups (18). In short, a FRT cell line was transfected with pFRT/lac-Zeo, and zeocin-resistant stable clones were selected as a Flp-In host cell line. The FRT/Flp-in cells were transfected with pcDNA5/FRT vector containing the CFTR variants that were constructed beforehand. FRT cells expressing the WT or CFTR variant cDNAs were first selected for hygromycin resistance in the presence of hygromycin-B (200 μg/mL). Hygromycin-resistant clones were then screened for β-gal activity; cells with the CFTR gene inserted into the FRT site disrupt lacZ expression and show negative β-gal activity. Finally, cells with negative β-gal expression were used to measure CFTR mRNA transcripts by real-time RT-PCR; G550W Clone 19 and G550X Clone 7 were selected to study the G550 variants based on having a level of mRNA expression similar to the WT-CFTR cell line. To stimulate readthrough, cells were treated with G418 or ELX-02 at indicated concentrations. The CFTR correctors tezacaftor (VX-661, 3 µM) and elexacaftor (VX-445, 3 µM), alone and in combination, were also tested in the presence or absence of G418 1,000 µg/mL (1,444 µM) or ELX-02 1,000 µg/mL (1,720 µM).

### Patch Recording and Data Analysis

Macroscopic and microscopic currents were recorded with the excised inside-out patch configuration, as previously described (19, 20). Patch pipettes were pulled and fire polished from Corning 8250 glass tubing with a tip resistance of 4–6 and 9–12 MΩ for macroscopic and microscopic recordings, respectively, when filled with recording solution. Pipette and bath solutions were identical and contained (in mM) 140 *N*-methyl-d-glucamine, 3 MgCl_2_, 1 EGTA, and 10 TES, adjusted to pH 7.3 with HCl. CFTR channels were activated by 1.5 mM Mg-ATP and 123 U/mL PKA (purified from recombinant *Escherichia coli* expressing the catalytic subunit of bovine PKA, 90% pure) in the cytosolic bath. Macroscopic currents were recorded with a ramp protocol (±80 mV) with a 10-s period and analog filtered at 20 Hz by using transient transfected HEK293 cells. Single-channel studies for WT- and G550W-CFTR were performed with the stably transfected FRT cells. Membrane patches were held at 60 mV for microscopic (unitary) current recording, and the current signals were analog filtered at 200 Hz and digitized at a sampling rate of 2 kHz. The channel open probability (*P*_o_) was analyzed as previously described (20). In brief, only records that contained fewer than eight simultaneous openings after CFTR potentiator (VX-770 and/or curcumin) addition were analyzed. *P*_o_ was estimated by dividing the *NP*_o_ product measured for that condition by the maximal number of simultaneous openings observed after CFTR potentiators (*N*). Mean burst durations (MBDs, in seconds) and interburst intervals (IBIs) were estimated for the same filtered multichannel records with the cycle time method (21), where *T* is the length of the channel record (in seconds):

(*1*) $$\mathrm{MBD=}\left(NP_{o}\right)T/\text{number of openings}$$

(*2*) $$\mathrm{IBI}=\left[\left(N-NP_{o}\right)T\right]/\left(\text{number of openings}-\text{1}\right)$$

Data acquisition and analysis were performed with pCLAMP 9.2 software (Axon Instruments). All patch-clamp experiments were performed at room temperature (∼21–23°C).

### Quantitative Real-Time PCR

Total RNA was extracted with the RNeasy Mini Kit (Qiagen) according to the manufacturer’s instructions. RNA concentration was quantitated with a NanoDrop (ThermoFisher). Quantitative real-time PCR was performed with the Applied Biosystems QuantStudio Real-Time PCR Detection System with TaqMan One-Step protocol and CFTR and GAPDH mRNA expression assay (CFTR assay ID: Hs00357011_m1, GAPDH assay ID: Rn01775763_g1) (ThermoFisher) (22, 23). Results were expressed as fold change compared to WT after the expression of human CFTR gene normalized to the reference human housekeeping GAPDH gene.

### Western Blot

HEK293 cells were washed with 1× PBS, harvested, and lysed with M-PER Mammalian Protein Extraction Reagent (Thermo Scientific, 78501) with protease inhibitors (Roche, 11873580001). Lysates were centrifuged at 14,000 *g* for 10 min at 4°C. Lysates were normalized for total protein concentration, reduced, separated by electrophoresis, and transferred to Immobilon-FL PVDF membrane (EMD Millipore). Membranes were blocked in 1× PBS with 5% milk (wt/vol) and 0.3% Tween 20 (vol/vol) overnight. The next day membranes were incubated with TurboGFP antibody (Pierce, 1:2,000), anti-hemagglutinin (HA) antibody (Covance Research, 1:2,000) and anti-tubulin antibody (DSHB, 1:2,000) followed by secondary antibodies (IRDye 680RD and IRDye 800CW, Li-Cor, 1:20,000). The blots were then imaged with the Li-Cor Odyssey CLx Infrared Imaging System (7).

CFTR protein expression in FRT cells was determined by Western blot as previously described (12). In brief, FRT cell lysates were normalized for protein concentration and separated by 4–15% gel electrophoresis. CFTR was detected with UNC596 monoclonal anti-CFTR antibody (University of North Carolina at Chapel Hill, 1:5,000), which has been used by us and other investigators for years (24–26); the loading control β-actin was detected by polyclonal anti-β-actin (Sigma no. A2066, dilution 1:2,000).

### Transepithelial Cell Conductance Measurements of FRT Cell Monolayers

FRT cells were seeded onto HTS-24 transwell plate inserts (Corning Inc.) at a density of 0.1 × 10^6^/0.33 cm^2^/insert and maintained for 4 days in FRT medium at 37°C with 5% CO_2_. Vehicle (0.2% DMSO), G418 (1,000 µg/mL = 1,444 µM) alone, ELX-02 (1,000 µg/mL = 1,720 µM) alone, tezacaftor (VX-661, 3 µM) alone, a mixture of elexacaftor (VX-445, 3 µM) and tezacaftor, and combinations were added to medium of FRT G550X or G542X cells on both basolateral and apical sides and incubated for 48 h before assay. Various doses of G418 (0–1,000 µg/mL) and ELX-02 (0–2,000 µg/mL) were used for dose-response studies in FRT G542X and G550X cells. Cell conductance (*G*_t_) was measured with a 24-channel voltage clamp (TECC-24; EP Design, Belgium) and mounted on a robotic platform (Yamaha, Japan) based on previously described methods (12, 27). After baseline conductance (*G*_t_) was recorded, forskolin (10 μM), ivacaftor (1 µM), and CFTR_Inh_-172 (10 μM; Selleckchem) were added sequentially to both basolateral and apical sides (28). Forskolin at concentrations ranging from 1 nM to 10 μM was used in the dose-response studies for WT and G550W FRT cells.

### Statistical Analysis

Patch-clamp data acquisition and analysis were performed with pCLAMP 9.2 software (Axon Instruments). FIS, PCR, Western blot densitometry, cell transepithelial electrical resistance (TEER), and cell *G*_t_ data were analyzed with GraphPad Prism 9.0 software. Student’s unpaired *t* test and ordinary one-way ANOVA with Tukey’s multiple comparison test were used for statistical analysis. *P* values < 0.05 were considered to indicate statistical significance.

## RESULTS

### ELX-02 Restores CFTR Function in G550X and G542X PDOs

**Figure 1.**
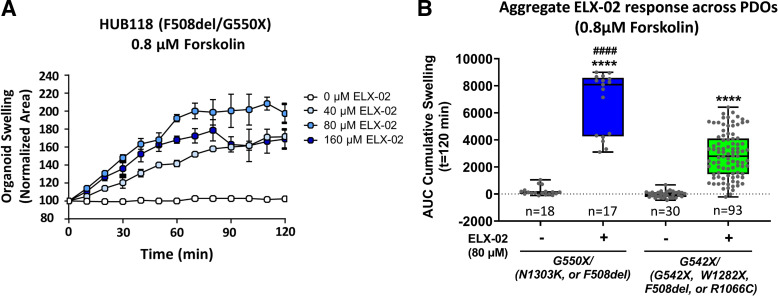
ELX-02 response across intestinal patient-derived intestinal organoids (PDOs) with at least 1 G550X or G542X allele. G550X or G542X PDOs were pretreated with vehicle or ELX-02 (40 µM, 80 µM, and 120 µM) for 48 h and then were imaged every 10 min up to 120 min after swelling induction with 0.8 µM forskolin. The average change in PDO size was tracked and represented as a percent change from baseline (100%), from which the area under the curve (AUC) was calculated. *A*: representative F508del/G550X organoids show an increase in swelling size over time in response to ELX-02 at a concentration of 80 µM. Data are expressed as means ± SD. *B*: organoid swelling size and AUC values generated from multiple independent experiments from each PDO were compiled to assess the impact of ELX-02 on CFTR activity in PDOs bearing at least 1 G550X allele (*n* = 6) or G542X allele (*n* = 5); the total number of organoids is indicated. AUC was calculated for the cumulative swelling observed for each organoid, and the results are shown in box and whiskers plots. One-way ANOVA with Sidak’s multiple comparison analysis revealed a significant effect of ELX-02. The response of ELX-02 in G550X PDOs is significantly higher than that in G542X PDOs. ****P < 0.0001 vs. vehicle, ####*P* < 0.0001 vs. ELX-02-treated G542X group. G542X control data in *B* from Ref. 13.

ELX-02 was evaluated in six intestinal PDOs bearing one G550X-CFTR mutation together with an F508del allele (*n* = 5) or an N1303K allele (*n* = 1). All six G550X PDOs responded to ELX-02 treatment, with swelling observed with 0.128 µM forskolin induction and further increased at higher forskolin induction levels (Supplemental Fig. S1). Dose dependence of the ELX-02 response was observed when a low forskolin concentration was used to activate swelling in three G550X/F508del PDOs (HUB84, HUB178, and HUB118). The remaining two G550X/F508del (HUB226, HUB213) and one G550X/N1303K (HUB130) PDO data lacked a clear ELX-02 dose-dependent response and suggest a plateau in swelling at the 0.8 µM and 2 µM forskolin concentrations. The preswelling of ELX-02-treated organoids before the addition of forskolin may be a potential factor, which could contribute to a ceiling effect that limited dose dependence. Crawford et al. (13) have demonstrated that ELX-02 produces significant readthrough of the G542X allele, leading to increased CFTR mRNA, protein, and protein function across five G542X PDOs, when the second CFTR allele was G542X, F508del, W1282X, R1066C-A, or R1066C-B, and the G542X/R1162C-A and G542X homozygous PDOs had better response to ELX-02 than G542X/W1282X, G542X/F508del, and G542X/R1162C-B PDOs. To compare the ELX-02 response in both G550X and G542X PDOs, vehicle- and 80 μM ELX-02-pretreated samples were further analyzed by organoid swelling over a 120-min period with 0.8 µM forskolin induction. The time course of swelling over 120 min in representative G550X organoids demonstrated a significant increase in swelling with ELX-02 treatment (Fig. 1*A*). The cumulative swelling response [area under the swelling curve (AUC) from 0 to 120 min] of ELX-02-treated G550X PDOs was significantly higher than that in the G542X PDOs (AUC: 6,773 ± 2,263 vs. 2,886 ± 1,679) (Fig. 1*B*), indicating that the G550X allele generated 2.3-fold more CFTR function in response to readthrough mediated by ELX-02.

### ELX-02 Induces Insertion of the Amino Acid Tryptophan in the G550X-CFTR Context

**Figure 2.**
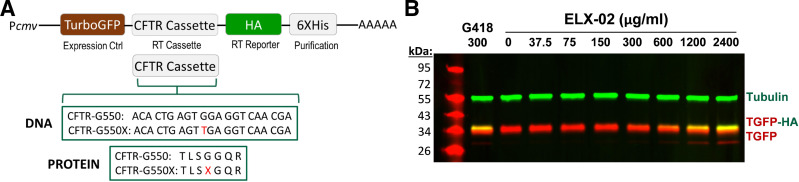
Determination of the amino acids inserted during G418- or ELX-02-mediated suppression of the G550X CFTR premature termination codon (PTC) in HEK293 cells. *A*: TurboGFP (TGFP) reporters were used to identify amino acids inserted at CFTR PTCs during readthrough. The G550X-CFTR reporter (and the CFTR context included) is shown. *B*: representative Western blot showing an increased amount of full-length protein [containing both TGFP and hemagglutinin (HA) tag] (yellow-green bands) compared with the TGFP protein lacking the HA tag (red bands) after 48 h of G418 (300 µg/mL) or ELX-02 (0–2,400 µg/mL) treatment in HEK293 cells stably transfected with the G550X reporter. Tubulin was used as a loading control.

We have previously identified three amino acids, Cys (C), Trp (W), and Arg (R), that are inserted into the G542X-CFTR PTC (a UGA) in HEK293 reporter cells treated with the aminoglycoside G418 (7). The G542R-, G542C-, and G542W-CFTR protein variants that are generated exhibit different levels of CFTR activity; none of these variants achieves the activity seen with WT-CFTR because of processing and functional abnormalities (8). To understand why the G550X allele, also a UGA, is more responsive to aminoglycoside-mediated readthrough than the G542X allele, we used a similar strategy as before to identify the amino acid(s) inserted in the G550X CFTR context after ELX-02 treatment. Previous studies have shown that local mRNA sequence context around a PTC acts as a primary determinant of readthrough efficiency (8, 29). In the present study, we used a TurboGFP (TGFP) reporter system with the local CFTR mRNA context surrounding the G550 position (3 codons upstream and downstream) into the readthrough cassette to generate a readthrough product. This reporter construct contains an upstream TGFP followed by the G550X-CFTR mutation and surrounding context; COOH-terminal HA and 6× polyhistidine (His) tags reside downstream (Fig. 2*A*). After PTC suppression, the full-length protein contains TGFP, along with HA and 6× His tags, whereas the prematurely terminated protein contains TGFP only. We treated HEK293 cells stably expressing the G550X TGFP construct with ELX-02 600 µg/mL (1,032 µM) for 48 h, using G418 300 µg/mL (433 µM) as a positive control. Western blotting confirmed an increased abundance of full-length protein containing both TurboGFP and HA tag (Fig. 2*B*). Assessment of readthrough products purified by affinity chromatography and subjected to MS/MS analysis (7, 8) revealed that tryptophan (W) was the sole amino acid inserted in the 550 position in response to either ELX-02 or G418 (Supplemental Fig. S2), a result that was dissimilar to the G542X-CFTR allele, where three amino acids (44% C, 36% W, and 20% R) were incorporated after G418-mediated suppression. Because the same PTC (UGA) was tested in both the G542X and G550X constructs and they differ only by the three codons upstream and three codons downstream of the PTC, our results indicate that the local sequence context surrounding a PTC can influence both the amino acid(s) inserted during readthrough and their relative proportions. This raised the possibility that the G550W-CFTR substitution in the readthrough product from ELX-02- or G418-treated cells may contribute to the high CFTR activity with the G550X allele.

### FRT Cells Expressing G550W-CFTR Exhibit Similar CFTR Protein Expression and Enhanced Function vs. WT-CFTR

**Figure 3.**
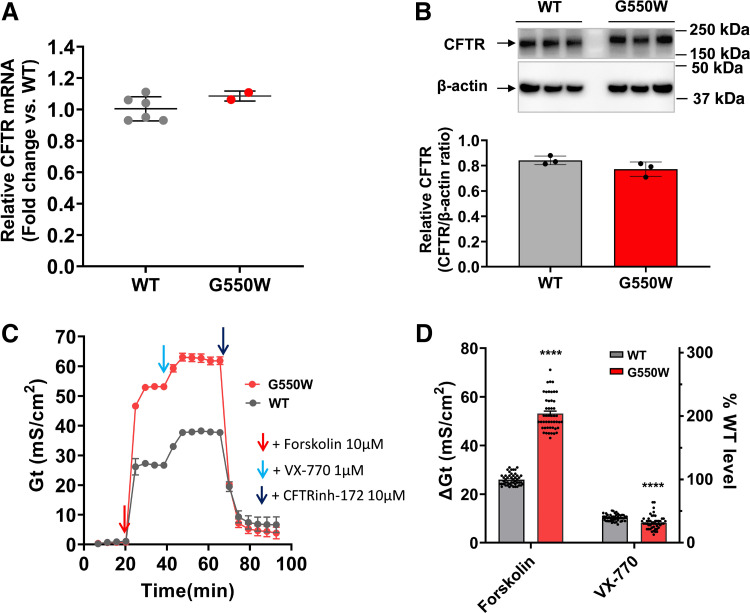
CFTR mRNA and protein expression and function in Fischer rat thyroid (FRT) cells expressing wild-type (WT)- vs. G550W-CFTR. *A*: WT-CFTR (*n* = 6) and G550W-CFTR (*n* = 2) mRNAs were similarly expressed in FRT cells (*n* = 2). *B*: CFTR protein expression was detected by Western blot. WT-CFTR (*n* = 3) and G550W-CFTR (*n* = 3) proteins were expressed at similar levels in FRT cells. *C* and *D*: tracings of cell conductance (*G*_t_) (*C*) and summary Δ*G*_t_ plots (*D*) of FRT cell monolayers expressing WT-CFTR or G550W-CFTR; CFTR function in FRT cells expressing G550W-CFTR was increased compared with WT-CFTR. The *G*_t_ of the FRT cell monolayers was measured with the transepithelial cell conductance (TECC) assay. CFTR activity was determined as the change from baseline transepithelial conductance (Δ*G*_t_) after the addition of forskolin (10 µM) and/or VX-770 (1 µM), CFTR_Inh_-172 (10 µM) was used to block CFTR-mediated *G*_t_ for confirmation. Data are shown as means ± SE of Δ*G*_t_; *n* = 48 per group, performed in 2 independent experiments. Student’s unpaired *t* test was used for statistical analysis between WT and G550W groups. ****P < 0.0001 vs. WT.

Mass spectrometry confirmed tryptophan (W) as the sole amino acid inserted in the G550X-CFTR PTC position in response to ELX-02 and G418 in HEK cells (Supplemental Fig. S2). However, as tryptophan differs from the amino acid that normally resides at codon 550, a glycine, it is unclear whether the resulting full-length CFTR variant protein is expressed or functions like the WT-CFTR protein. To address this question, we generated FRT cell lines stably expressing G550W-CFTR that had CFTR expression similar to WT-CFTR (30) and then compared CFTR expression and function to cells expressing WT-CFTR in response to pharmacological treatment with aminoglycosides. Quantitation of CFTR mRNA showed that its abundance in the G550X- and WT-CFTR cell lines were similar (Fig. 3*A*). Western blot revealed that the level of CFTR protein expression in G550W FRT cells (CFTR-to-β-actin ratio: 0.77 ± 0.06) was also similar to that seen in WT cells (0.81 ± 0.03, *P* > 0.05) (Fig. 3*B*). For assessment of CFTR function by transepithelial cell conductance (TECC) assay, a cAMP agonist, forskolin, was used to activate CFTR channel opening, followed by the addition of the CFTR potentiator VX-770 (ivacaftor) and then the CFTR inhibitor CFTRinh-172. VX-770 binds to CFTR protein at the cell surface and promotes channel opening, enabling chloride transport and thereby regulating water movement across the cell membrane (18, 31). VX-770 has been used for a broad range of CFTR mutations with gating abnormalities. Compared with the WT-CFTR control, FRT cells expressing G550W-CFTR exhibited significantly increased forskolin-activated Cl^−^ conductance (53.1 ± 6.9 vs. 26.0 ± 2.3 mS/cm^2^, *P* < 0.0001), reaching about two times the level of WT-CFTR (Fig. 3, *C* and *D*). Interestingly, G550W FRT cells exhibited significantly reduced VX-770-activated Cl^−^ conductance compared with WT after forskolin activation (8.1 ± 0.5 vs. 10.3 ± 0.2 mS/cm^2^, *P* < 0.0001) (Fig. 3, *C* and *D*), suggesting that G550W-CFTR reached higher activation level than WT under same condition. Taken together, we conclude that the insertion of the amino acid tryptophan (W) at the G550X-CFTR PTC during aminoglycoside-mediated readthrough generates a variant CFTR protein with enhanced function independent of processing efficiency.

### G550W-CFTR Increases Forskolin Response, PKA Sensitivity, and Channel Open Probability

Forskolin increases intracellular cAMP levels through activation of adenylyl cyclase in various cell types. Extracellular application of micromolar concentrations of forskolin (0.5–10 μM) elicits an intracellular increase of cAMP that is sufficient to activate PKA, with the consequent gating of the CFTR channel mediated by ATP binding that in turn leads to activation of CFTR (32, 33).

**Figure 4.**
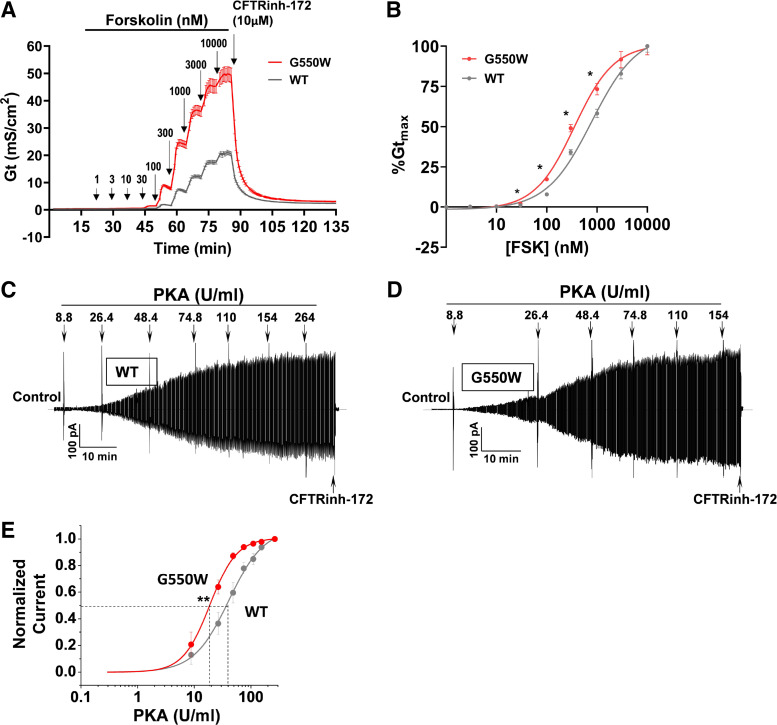
G550W-CFTR shows increased forskolin response and PKA sensitivity compared with wild-type (WT)-CFTR. *A*: tracings of the conductance (*G*_t_) of Fischer rat thyroid (FRT) cell monolayers by transepithelial cell conductance (TECC) assay show CFTR activity in response to different doses (the additions are of each new dose in nM) of forskolin. Data are shown as means ± SD of *G*_t_; *n* = 12 per group. *B*: comparison of forskolin (FSK) dose-response curve between G550W-CFTR (red) and WT (gray). G550W-CFTR is more sensitive to forskolin than WT-CFTR. Each symbol is the mean ± SE of the % maximum conductance (*G*_t max_) at the corresponding forskolin concentration for each group. *P < 0.05 vs. WT. *C* and *D*: patch-clamp macroscopic current records from HEK-293 cells expressing WT (*C*)- or G550W (*D*)-CFTR show PKA titration. CFTR currents were evoked by a ramp protocol (±80 mV). Control currents were recorded in the presence of 1.5 mM Mg-ATP only followed by addition of different doses of PKA to the bath where indicated. *E*: comparison of PKA dose-response curve between WT (gray)- and G550W (red)-CFTR. Each symbol is the mean ± SE for G550W (*n* = 6) and WT (*n* = 7) experiments. Data were fit to the Michaelis–Menten equation; *K*_m_ = 19.6 ± 2.9 and 49.5 ± 5.9 U/mL for G550W and WT, respectively. **P < 0.01 by *t* test.

**Figure 5.**
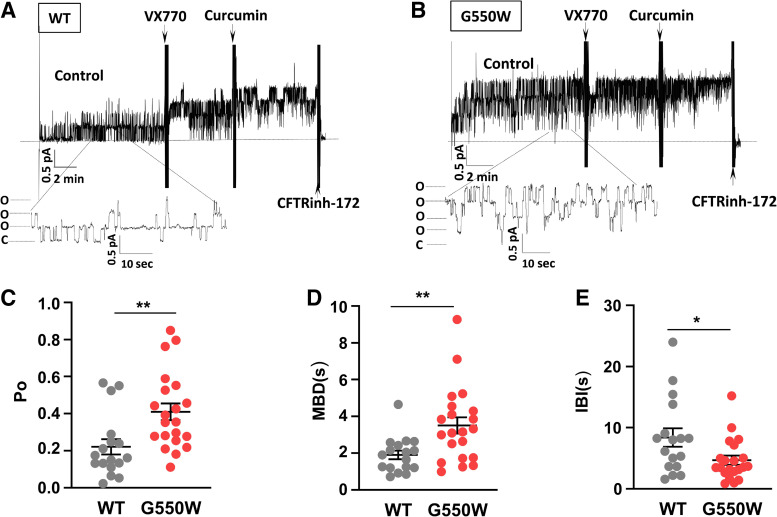
G550W-CFTR increases channel open probability. *A* and *B*: unitary channel records for inside-out patch excised from Fischer rat thyroid (FRT) cell stably expressing wild-type (WT)- and G550W-CFTR. CFTR channels were activated in the presence of 1.5 mM Mg-ATP and 123 U/mL PKA. Holding potential was +60 mV. *Insets*, unitary currents from WT and G550W-CFTR under control conditions. *C–E*: comparison of estimated mean open probability (*P*_o_), mean burst duration (MBD), and mean interburst interval (IBI) for WT- and G550W-CFTR channels. Error bars represent SEs; *n* values as indicated. *P < 0.05 and **P < 0.01 by *t* test.

To further examine how G550W variant promotes CFTR activation, we first tested the idea of whether G550W-CFTR channel activity surpassing WT levels in FRT cells is due to increased forskolin sensitivity. Using TECC assay, we examined changes in cell conductance in response to various doses of forskolin (1 nM to 10 µM) in FRT cells stably expressing WT- or G550W-CFTR. G550W-CFTR demonstrated an increased forskolin response compared with WT-CFTR (Fig. 4*A*) as well as exhibiting greater sensitivity to forskolin (FSK EC_50_: 354 nM vs. 783 nM) (Fig. 4*B*), suggesting enhanced cAMP associated phosphorylation of G550W variant. To further confirm this, we then performed PKA titration by patch analysis. Figure 4, *C–E*, shows PKA titrations and mean dose-response curves for both WT and G550W-CFTR. Under the control condition, but in the absence of PKA, macroscopic currents from both WT and G550W are difficult to detect, which is consistent with the requirement of phosphorylation for CFTR channel activation. Channel activity gradually appeared when a low dose of PKA (e.g., 8.8 U/mL) was added in the bath. As shown in Fig. 4, *C* and *D*, G550W-CFTR could reach high activity at a relatively low dose of PKA (e.g., between 8.8 and 48.4 U/mL) compared with WT. G550W variant significantly shifted the PKA dose-response curve to the left with an EC_50_ of 19.6 ± 2.9 U/mL compared with 49.5 ± 5.9 U/mL for WT (Fig. 4*E*), suggesting that the G550W variant promotes channel activity by increasing PKA sensitivity, although G550W slightly increases ATP sensitivity compared with WT (Supplemental Fig. S3). In addition, to examine whether G550W variant alters channel gating (e.g., open probability), we then performed unitary channel analysis. Figure 5 shows representative unitary channel recordings of WT- and G550W-CFTR and mean data of channel open probability (*P*_o_), mean burst duration (MBD), and mean interburst interval (IBI). By measuring the current amplitude of single channels, we found that the G550W variant displays unitary current (≅0.4) or unitary conductance (≅0.7 pS) similar to WT, indicative of less alternation of G550W on CFTR pore. In contrast, as shown in Fig. 5, *C–E*, G550W variant significantly increased channel *P*_o_ (0.41 ± 0.05 vs. 0.22 ± 0.04, *P* < 0.01) and MBD (3.5 ± 0.44 vs. 1.9 ± 0.23, *P* < 0.01) and decreased IBI (4.69 ± 0.74 vs. 8.39 ± 1.49, *P* < 0.05) compared with WT, indicating that the G550W variant enhances channel activity not only by increasing PKA sensitivity but also by promoting channel gating. Together, these results support that the G550W variant is a gain-of-function (GOF) mutation and predict that the insertion of tryptophan at the G550X-CFTR PTC during aminoglycoside-mediated readthrough could increase CFTR function.

### CFTR Activity Is Elevated in FRT Cells Expressing G550X- vs. G542X-CFTR in Response to ELX-02

**Figure 6.**
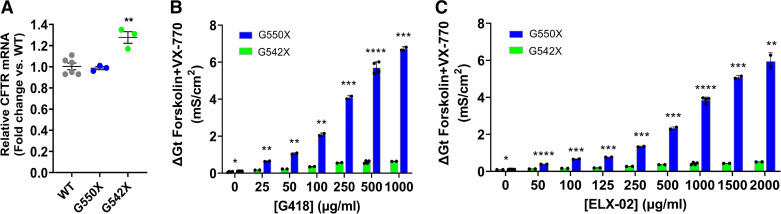
CFTR activity is rescued by aminoglycosides in Fischer rat thyroid (FRT) cells expressing G550X- or G542X-CFTR cDNAs. *A*: FRT cells stably expressing G550X- or G542X-CFTR cDNAs were generated and compared to cells expressing wild-type (WT) CFTR. CFTR mRNA level in FRT cells expressing G550X was similar, while a G542X clone had a higher CFTR mRNA expression level. Ordinary 1-way ANOVA with Tukey’s multiple comparison test were used for statistical analysis. **P < 0.01 vs. WT or G550X. *B* and *C*: FRT cells stably expressing G550X- or G542X- CFTR cDNAs were pretreated with various concentrations of G418 (*B*) or ELX-02 (*C*) for 48 h before cell conductance measurement by transepithelial cell conductance (TECC) assay. CFTR activity is expressed as the total change of cell conductance (Δ*G*_t_) after forskolin and VX-770 stimulations from baseline. Each symbol is the mean ± SD of Δ*G*_t_ corresponding to G418 or ELX-02 concentration for each group (*n* = 2–4/concentration, from 2 independent experiments). *C*: comparison of G418 or ELX-02 dose response between G550X-CFTR and G542X-CFTR. Multiple unpaired *t* tests were used for statistical analysis. *P < 0.05, **P < 0.01, ***P < 0.001, and ****P < 0.0001 vs. G542X group.

We further evaluated the dose response of ELX-02 on CFTR activity in FRT cells stably expressing G550X-CFTR or G542X-CFTR, using the TECC assay. Before the CFTR function measurement, we generated FRT cell lines stably expressing a G550X CFTR cDNA. Compared with WT-CFTR, the CFTR mRNA level in FRT cells expressing G550X (1.06 ± 0.03 fold change relative to WT-CFTR) was similar (Fig. 6*A*). In contrast, the FRT cells expressing the G542X cDNA had slightly higher CFTR mRNA expression level, although unlikely to be biologically relevant (1.27 ± 0.09, *P* < 0.01) (Fig. 6*A*). Cell lines were treated with ELX-02 at a dose range of 0–2,000 μg/mL or with G418 at a range of 0–1,000 μg/mL for 48 h before cell conductance measurements by the TECC assay. At the maximum G418 dose of 1,000 µg/mL (1,444 µM), significant increases in CFTR activity were observed in both cell lines, although levels with the G550X construct were 10.5-fold higher than with G542X (6.73 ± 0.11 vs. 0.64 ± 0.07 mS/cm^2^, *P* < 0.001), reaching 18.5% and 1.8% of WT, respectively (Fig. 6*B*). Similarly, ELX-02 at a concentration of 1,000 µg/mL (1,720 µM) elicited 9.2-fold higher CFTR activity in FRT cells with the G550X construct than with G542X (3.86 ± 0.16 vs. 0.42 ± 0.06 mS/cm^2^, *P* < 0.0001), reaching 10.6% and 1.3% of WT levels, respectively (Fig. 6*C*). Significant decreases of monolayer cell resistance upon treatment with ELX-02 (1,000 µg/mL = 1,720 µM) (2,467.3 ± 499.9 Ω·cm^2^) were not observed compared with G418 (1,000 µg/mL = 1,444 µM) (1,000.7 ± 70.7 Ω·cm^2^, *P* < 0.05), Furthermore, ELX-02 alone at high dose (2,000 µg/mL = 3,440 µM) did not reduce monolayer cell resistance (1,951.5 ± 283.6 Ω·cm^2^ vs. vehicle 2,379 ± 324.5 Ω·cm^2^) measured by TECC assay in G550X FRT cells, suggesting that ELX-02 is less toxic than G418, consistent with prior reports (11, 12). These findings indicate that G550X-CFTR is much more susceptible to restoration of CFTR activity by ELX-02 and G418 in a dose-dependent fashion compared with G542X-CFTR.

### CFTR Correctors Augment CFTR Protein Expression and Function in FRT Cells Expressing G550X- or G542X-CFTR after Aminoglycoside Treatment

**Figure 7.**
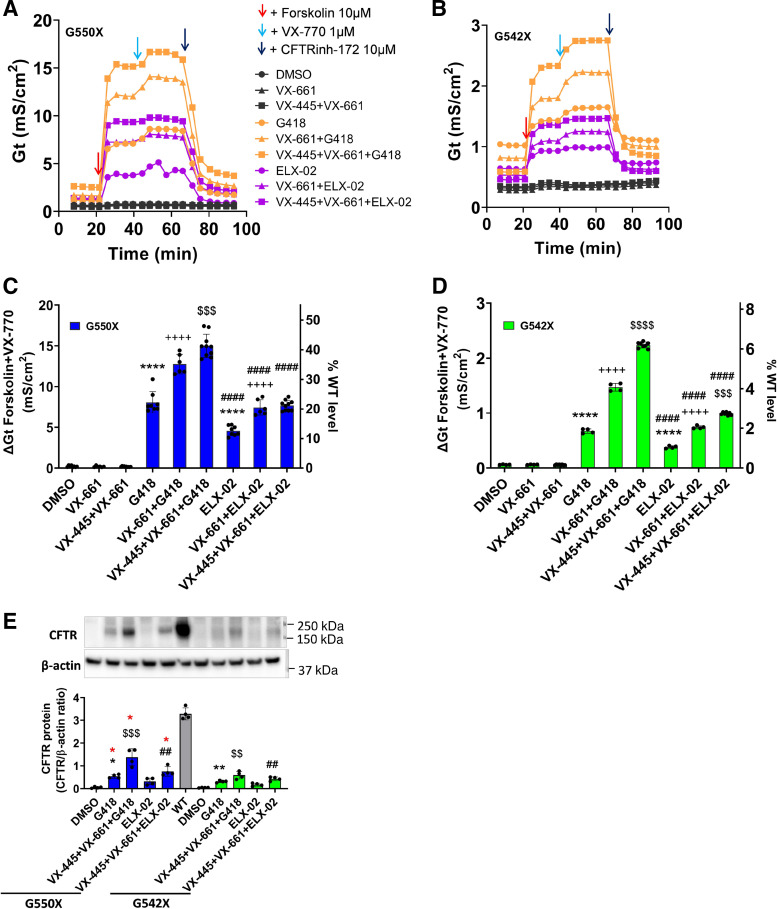
CFTR activity in Fischer rat thyroid (FRT) cells expressing G550X- or G542X-CFTR and cotreated with CFTR correctors and aminoglycosides. *A–D*: representative cell conductance (*G*_t_) tracings, summary change of *G*_t_ (Δ*G*_t_), and percentage of wild-type (WT) level plots of FRT cell monolayers expressing CFTR-G550X (*A* and *C*) or CFTR-G542X (*B* and *D*). Both cell lines were pretreated for 48 h with CFTR correctors [VX-661 (3 µM) or VX-445 (3 µM) + VX-661 (3 µM)] alone or in combination with G418 (1,000 µg/mL) or ELX-02 (1,000 µg/mL), which was followed by acute addition of forskolin, VX-770, and CFTRInh-172. CFTR function significantly increased in response to aminoglycosides and CFTR correctors. ****P < 0.0001 vs. vehicle; ++++*P* < 0.0001 vs. corresponding group without VX-661; $$$*P* < 0.001 and $$$$*P* < 0.0001 vs. corresponding group without VX-445; ####*P* < 0.0001 vs. corresponding group with G418. After the transepithelial cell conductance (TECC) assay, the cell samples were collected for CFTR protein detection by Western blot. *E*: blot images and their corresponding densitometry plots (*n* = 4) represented as the fold change of CFTR protein after normalization to β-actin compared with vehicle treatment. FRT WT was added as a control. Quantitation of CFTR protein expression showed a increase in G550X-CFTR vs. G542X-CFTR protein in response to aminoglycosides and CFTR correctors. *P < 0.05 and **P < 0.01 vs. vehicle; $$*P* < 0.01 and $$$*P* < 0.001 vs. G418; ##*P* < 0.01 vs. ELX-02 in either G550X or G542X group. Red *P < 0.05 vs. G542 corresponding groups. Ordinary 1-way ANOVA with Tukey’s multiple comparison test were used for statistical analysis.

We next evaluated whether CFTR correctors can further augment the effect of G418 or ELX-02 on CFTR protein expression and function in FRT cells expressing G550X- or G542X-CFTR. As single agents, G418 (1,000 µg/mL = 1,444 µM) (8.08 ± 1.29 mS/cm^2^, *P* < 0.0001) and ELX-02 (1,000 µg/mL = 1,720 µM) (4.57 ± 0.60 mS/cm^2^, *P* < 0.0001) significantly increased CFTR activity in G550X-CFTR FRT cells compared with vehicle (0.21 ± 0.09 mS/cm^2^), reaching 22.2% and 12.6% of activity observed in cells expressing WT-CFTR (36.3 ± 3.0 mS/cm^2^), respectively (Fig. 7, *A* and *C*). CFTR activity was notably lower in G542X-CFTR FRT cells treated with G418 (0.67 ± 0.04, *P* < 0.0001) or ELX-02 (0.38 ± 0.02, *P* < 0.0001) alone, reaching only 1.8% and 1.0% of WT level, respectively (Fig. 7, *B* and *D*). These results corroborate earlier data that CFTR rescued by aminoglycoside-mediated readthrough of the G550X allele is more active than CFTR rescued by readthrough of the G542X allele.

The CFTR corrector VX-661, alone or in combination with VX-445, did not improve CFTR function in G550X (0.19 ± 0.09; 0.17 ± 0.04) (Fig. 7*C*) or G542X (0.06 ± 0.01; 0.06 ± 0.01) (Fig. 7*D*) FRT cells compared with vehicle (0.21 ± 0.09), suggesting that negligible full-length CFTR might be expressed without the presence of a readthrough agent and that the CFTR protein fragments terminated at positions G550 and G542 are not functional even when the cells were treated with both correctors.

However, when VX-661 was combined with G418 (1,000 µg/mL = 1,444 µM) (12.8 ± 1.21) or ELX-02 (1,000 µg/mL = 1,720 µM) (7.44 ± 0.94) in G550X FRT cells, CFTR activity reached 35.3% and 20.5% of WT, respectively (Fig. 7*C*); a benefit with the combination of VX-661 and an aminoglycoside was also observed in G542X FRT cells (VX-661 + G418, 1.47 ± 0.07 and VX-661 + ELX-02, 0.74 ± 0.04), but only 4.0% or 2.0% of WT level was reached in G542X cells, respectively (Fig. 7*D*). Moreover, the combined addition of CFTR correctors VX-445 + VX-661 further augmented CFTR activity in G550X FRT cells in the presence of G418 (15.00 ± 1.42) or ELX-02 (7.69 ± 0.64), reaching 41.3% and 21.2% of the WT level, respectively, levels approximately twofold greater than seen with G418 or ELX-02 alone (Fig. 7*C*). In contrast, CFTR activity in G542X FRT cells treated with VX-445 + VX-661 and G418 (2.24 ± 0.05) or ELX-02 (0.99 ± 0.03) only reached 6.2% and 2.7% of WT level, respectively (Fig. 7*D*).

To further confirm that restoration of CFTR function in G550X and G542X FRT cells was accompanied by the expression of full-length, mature CFTR protein, we conducted Western blotting of cell monolayers after TECC assay following treatment with aminoglycosides and CFTR correctors. Compared with vehicle, CFTR protein expression was significantly increased in G418-treated G550X (CFTR-to- β-actin ratio: 0.54 ± 0.08 vs. vehicle 0.05 ± 0.03; *P* < 0.05) and G542X (0.31 ± 0.05 vs. vehicle 0.04 ± 0.01; *P* < 0.05) FRT cells, whereas CFTR protein expression in ELX-02-treated G550X (0.32 ± 0.15) or G542X (0.18 ± 0.07) FRT cells was minimally increased relative to vehicle-treated cells (Fig. 7*E*). We also found that the addition of the CFTR correctors VX-445 + VX-661 to G418- or ELX-02 treated cells further augmented CFTR expression in G550X FRT cells treated with G418 (1.37 ± 0.37 vs. 0.54 ± 0.08 G418 alone) or ELX-02 (0.75 ± 0.21 vs. 0.32 ± 0.15 ELX-02 alone). Corrector treatment also enhanced CFTR activity in G542X FRT cells treated with G418 (0.59 ± 0.16 vs. 0.31 ± 0.05 G418 alone) or ELX-02 (0.43 ± 0.09 vs. 0.18 ± 0.07 ELX-02 alone) (Fig. 7*E*). These data suggest that the CFTR protein produced from the G550X FRT cells was much more active than the protein produced from the G542X FRT cells. Overall, these results indicate that the CFTR generated via readthrough of G550X-CFTR was more active than the CFTR rescued by aminoglycoside-mediated readthrough of the G542X-CFTR allele and that the function of variant CFTR proteins produced by suppression of G550X CFTR can be significantly enhanced by cotreatment with clinically approved CFTR correctors VX-445 + VX-661.

## DISCUSSION

ELX-02 (NB124) is a eukaryotic ribosomal selective glycoside that binds to the ribosomal decoding center and reduces proofreading of the A site. This increases the frequency that near-cognate tRNAs become accommodated at a PTC, resulting in the generation of a full-length protein (34). Traditional aminoglycosides such as gentamicin were shown to rescue partial CFTR function in CF patients who carry a nonsense mutation (35). However, because of off-target effects that result in ototoxicity and nephrotoxicity, aminoglycosides cannot be administered long term as a nonsense suppression agent. A medicinal chemistry team reasoned that because a component of aminoglycoside toxicity stems from their interactions with mitochondrial ribosomes, generating designer aminoglycosides that have greater affinity for cytoplasmic ribosomes would reduce their toxicity and enhance their ability to promote readthrough at PTCs (10, 36). ELX-02, which shows reduced toxicity while retaining significant readthrough efficacy compared with traditional aminoglycosides such as geneticin (G418), is the result of this work. Our previous study showed that ELX-02 can partially restore CFTR function in FRT monolayers expressing G542X-CFTR in a dose-dependent manner. It can also partially rescue CFTR function in CF primary HBE cells derived from a G542X/F508del donor and rescue CFTR function in CFTR^−/−^ mice expressing a human G542X-CFTR transgene (12). The recent phase 2 clinical trials for efficacy of ELX-02 failed in CF subjects with the G542X mutation on one or both alleles. The major reason may be too low ELX-02 exposure in the lung. [Eloxx Pharmaceuticals Reports Topline Results from Phase 2 Combination Clinical Trial of ELX-02 in Class 1 Cystic Fibrosis (CF) Patients; source: Eloxx Pharmaceuticals, Inc., September 22, 2022]. Further research is needed to improve ELX-02 exposure in the lung, particularly airway epithelial cells, where CFTR expression is relevant to lung physiology.

In the present study, the efficacy of ELX-02 was initially tested in CF PDOs carrying either the G550X- or G542X-CFTR nonsense mutation. The PDO response to ELX-02 was evaluated by performing forskolin-induced swelling (FIS) assays, which are dependent on CFTR channel activity. The level of activity observed with low concentrations of both ELX-02 and forskolin stimulation in addition to evidence of preswelling before forskolin addition support that the G550X allele is more responsive compared with the G542X allele. Quantification of the forskolin-induced swelling likely underestimated the effect of ELX-02 in this genotype because of the preswollen state of the organoids before forskolin addition. The reason that more CFTR function could be generated from the G550X allele than G542X could be *1*) increased readthrough frequency at the PTC or *2*) increased CFTR expression and/or function due to the identity/frequency of amino acids incorporated at the PTC. Because both G550X and G542X generate a UGA termination codon, the sequence context is likely responsible for the differences observed. However, the difference in readthrough frequency at the two contexts could not account for the large difference in CFTR function observed. We therefore examined the amino acids that become inserted at the PTC in both the G550X and G542X contexts when readthrough is generated with ELX-02.

Tryptophan was the only amino acid found to be inserted at the G550X-CFTR context during ELX-02-mediated readthrough. This result differs for the G542X context, where three near-cognate amino acids [arginine (R), cysteine (C), and tryptophan (W)] were inserted in the G542X-CFTR context during readthrough with ELX-02, which is in agreement with previous reports that examined amino acid incorporation at G542X with ataluren (PTC124) and G418 (7, 8). Although G550X and G542X PTC mutations both form UGA stop codons, the local context surrounding the UGA stop codon (as modeled by 3 codons upstream and downstream of the original CFTR mutation) is different. The increased CFTR activity obtained from the G550W cell line indicates that a UGA nonsense mutation can respond differently to translational readthrough agents as a function of the surrounding sequence context. Our results demonstrate that the local PTC sequence context plays an important role not only in the frequency of PTC suppression but also in the identity of amino acids incorporated at a PTC, which can have significant effects on the resulting protein expression or function (6).

Because the amino acid tryptophan (W) inserted at G550X-CFTR during aminoglycoside-mediated readthrough is different from the glycine amino acid normally expressed at that position in the WT-CFTR protein, the resulting G550W-CFTR protein could have altered processing and function compared with the WT-CFTR protein. However, we found that the FRTs stably expressing WT- and G550W-CFTR have similar CFTR expression and maturation, suggesting that the insertion of tryptophan at the G550X PTC augments CFTR function without altering CFTR abundance, processing, or trafficking. Evaluation of CFTR function showed that FRT cells expressing G550W-CFTR exhibited significantly increased forskolin activated Cl^−^ conductance, reaching about twice the WT level. These results are different from the G542-CFTR variants (G542R, G542C, and G542W) generated from readthrough of the G542X PTC, where among the three variants, CFTR activity in FRT cells stably expressing G542R and G542C showed ∼40–70% of WT level measured by short-circuit current (*I*_sc_), which correlates well with less full-length CFTR band C. The CFTR activity in G542W FRT cells only reached ∼10% of the WT level, consistent with little or no band C observed in the Western blot. Taken together, these results demonstrate that CFTR variants resulting from readthrough retain variable but significant functionality (7, 8). The amino acids inserted during readthrough at different CFTR PTCs can have significant effects on CFTR processing and function.

To further understand why G550W-CFTR has significantly better activity than WT-CFTR, we investigated the dose response for forskolin activation of G550W- and WT-CFTR stably expressed in FRT cells. The dose-response curve for activation by forskolin showed a remarkable increase in sensitivity for FRT G550W-CFTR relative to WT-CFTR over the entire concentration range tested (1 nM to 10 µM). The multichannel macropatch clamp found that G550W-CFTR largely increased PKA sensitivity by showing a significant shift of the PKA dose-response curve compared with WT-CFTR, although a small shift of the ATP dose-response curve was seen. These results indicate that G550W significantly increases forskolin and PKA sensitivity, leading to enhanced CFTR channel activity.

The unitary channel patch-clamp studies found that G550W-CFTR channel had increased open probability and mean burst duration (MBD), whereas mean interburst interval (IBI) was decreased compared with WT-CFTR. Our findings demonstrate that the increase in sensitivity to forskolin and PKA activation in G550W-CFTR is due to the intrinsic property of the G550W channel rather than being a secondary cooperative interaction among an increased number of mutant channels at the plasma membrane. Our findings indicate that the insertion of tryptophan (W) at the G550X PTC upon readthrough enhances the function of the resulting CFTR readthrough protein, rescuing more CFTR function from the CFTR variant generated from the G550X allele.

The enhancement of activity from the G550W variant protein makes sense when considering the structure of CFTR. CFTR is a unique member of the ATP-binding cassette transporter superfamily that forms an epithelial anion channel with complex regulation (37). Its TMDs form the channel pore through which anions flow along their electrochemical gradient, whereas NBDs control channel gating by binding and hydrolyzing ATP (38–40). The regulatory domain (RD) has multiple consensus phosphorylation sites (37), which promotes conformational changes necessary for channel functioning through protein kinase A (PKA) or protein kinase C (PKC) phosphorylation (41). Maximal phosphorylation of PKA sites in the regulatory domain controls the channel bursting rate and open probability (*P*_o_) of CFTR WT channels by increasing the apparent affinity of NBDs for ATP (42, 43). In vivo, CFTR is modified by different levels of phosphorylation, resulting in channels with correspondingly different biophysical characteristics (37, 40, 44). Gly-550 is a conserved residue within the signature NBD1 motif of ABC transporters (LSGGQ), including those with high homology to human CFTR (45). The functional importance of the LSGGQ region of CFTR NBD1 has been supported by previous mutational analysis describing processing and functional defects associated with amino acid substitutions in the conserved motif (46). Several studies have shown that the revertant G550E mutation can rescue processing and functional defects of the CFTR DeltaF508 (46–48). Deletion of a phenylalanine at amino acid position 508 (ΔF508) in the first nucleotide binding domain (NBD1) is the most prevalent CF-causing mutation and results in defective protein processing and reduced CFTR function, leading to chloride impermeability in CF epithelia and heterologous systems. The G550E mutation represents a nonconservative introduction of a negatively charged Glu residue, changing the LSGGQ core consensus signature sequence of NBD1 to LSEGQ. The G550E mutation increased the sensitivity of CFTR DeltaF508 and wild-type CFTR to activation by cAMP agonists, indicating that DeltaF508 defect can be significantly rescued by second-site mutations in the NBD1 region that includes the LSGGQ consensus motif (46). Roxo-Rosa et al. (47) found that G550E restored F508del-CFTR activity to WT-CFTR levels by altering the duration of channel openings and closings. Compared with F508del CFTR, the CF mutant G551D-CFTR reduces severely ATP-dependent channel gating without altering protein processing and had no effect on NBD1 dimerization (45). Sheppard’s group (48) found that the revertant G550E had marked effects on G551D-CFTR channel gating, strongly increasing opening frequency without altering the expression and maturation of G551D-CFTR protein. However, G550E did not restore single-channel activity to WT levels. All of these findings demonstrate that G550E has direct effects on CFTR structure but its action on CFTR processing and channel function is CF mutation specific. The G550W change inserted during G550X PTC suppression is located in the same position of the LSGGQ consensus motif as G550E. The molecular weight of the aromatic amino acid Trp (W) (mol wt: 240.6) with no charge is bigger than the negatively charged, acidic amino acid Glu (E) (mol wt: 147.1); these properties likely contribute to the unique properties of the G550W-CFTR variant protein.

To date, readthrough monotherapy has not been sufficient to restore the amount of CFTR function needed (20–30% of WT-CFTR function) to alleviate most CF clinical symptoms. A combination of readthrough drugs with other strategies may be needed. In the CF PDO model, de Poel et al. (14) demonstrated that the combination of ELX-02, the NMD inhibitor SMG1i, the correctors VX-445 and VX-661, and the potentiator VX-770 can significantly rescue CFTR function in organoids homozygous for W1282X-CFTR or homo-/heterozygous for G542X-CFTR. The organoid swelling obtained with the combination of pharmacotherapies was higher than the mean swelling of three VX-809/VX-770-rescued F508del/F508del organoid cultures, suggesting a level of rescue approaching clinical relevance that is comparable to the rescue of F508del/F508del organoids with VX-770/VX-809. However, efficacy varied between genotypes as well as within genotypes, suggesting that strong pharmacological rescue of PTCs may require a combination of drugs that target RT, NMD, and protein function (14). In the FRT cell lines, CFTR cDNAs were used that are not subject to splicing and therefore are not subject to classical, exon junction complex (EJC)-mediated NMD. Consequently, the expression of these cDNA constructs allowed us to correlate CFTR function directly with PTC suppression (12).

The CFTR potentiator ivacaftor (VX-770) has been approved for clinical use for the treatment of CF patients carrying the CFTR-G551D mutation (18). Recently, in vitro studies have demonstrated that the CFTR modulator TRIKAFTA [consisting of the correctors elexacaftor (VX-445) and tezacaftor (VX-661) and the potentiator ivacaftor (VX-770)] improves CFTR cellular processing and trafficking, and it has been approved for patients with CF aged 6 yr and older who have at least one F508del mutation in the CFTR gene or other approved mutations. We found that VX-661 alone or in combination with VX-445 did not improve CFTR function in FRT cells expressing G550X-CFTR or G542X-CFTR, indicating that the CFTR protein fragments terminated at positions G550 or G542 were not functional even when the cells were treated with correctors. However, in FRT cells stably expressing G550X CFTR, coadministration of one corrector, VX-661, with G418 or ELX-02 significantly enhanced CFTR activity, reaching 35.3% and 20.5%, respectively, of the WT level. Consistent with the functional data, we also found that coadministration of CFTR correctors VX-445 + VX-661 with G418 or ELX-02 further augmented CFTR protein expression levels in FRT cells expressing G550X-CFTR or G542X-CFTR. Addition of CFTR correctors VX-445 + VX-661 to G418 or ELX-02 treatment regimens also augmented CFTR activity, reaching 41.3% and 21.2%, respectively, of WT level. CFTR activity in FRT cells expressing G542X-CFTR with the same treatment combinations was <10% of WT. Variant CFTR proteins produced from readthrough likely escape from the endoplasmic reticulum (ER) after VX-661 or VX-445 + VX-661 treatment, transit to the cell surface, and undergo channel activation. Acute administration of the potentiator VX-770 further increased CFTR activity by increasing the gating of CFTR protein at the cell surface (31, 49, 50). These data demonstrate that the combination of CFTR correctors with a CFTR potentiator can significantly enhance the function of CFTR proteins generated by PTC suppression.

Taken together, the results described above suggest the G550W-CFTR variant generated from aminoglycoside-mediated readthrough of the G550X-CFTR allele was much more highly functional than CFTR variants restored from readthrough of the CFTR G542X allele. The expression and function of the G550W-CFTR variant protein could be significantly enhanced by cotreatment with clinically approved CFTR correctors VX-445 + VX-661. G550X-CFTR may be a particularly sensitive target for translational readthrough therapy and may be emblematic of a new class of “superresponder” readthrough variants. These data also present a systematic approach to examine individual nonsense mutations in CFTR and how they can be assessed in response to multimodal therapies.

## DATA AVAILABILITY

Data will be made available upon reasonable request.

## SUPPLEMENTAL DATA

10.6084/m9.figshare.22335733.v1 Supplemental Figs. S1–S3: https://doi.org/10.6084/m9.figshare.22335733.v1.

## GRANTS

This work was partially supported by a research contract from Eloxx Pharmaceuticals, Inc. to the University of Alabama at Birmingham (UAB) (S.M.R., UAB OSP# 000528460), NIH P30 P30DK072482 (to S.M.R. and D.M.B.), and the Cystic Fibrosis Foundation.

## DISCLOSURES

The work presented in this publication was partially funded by a research contract from Eloxx Pharmaceuticals, Inc. to UAB (S. M. Rowe, UAB OSP# 000528460). Please note that UAB had completed the research on this contract, including identifying the key findings, and exhausted the contract’s funds before August 2022. S. Aghamohammadzadeh is an employee of Eloxx Pharmaceuticals, Inc. None of the other authors has any conflicts of interest, financial or otherwise, to disclose.

## AUTHOR CONTRIBUTIONS

D.M.B. and S.M.R. conceived and designed research; J.C., K.T., L.F., W.W., S.A., H.W., and L.T. performed experiments; J.C., K.T., L.F., W.W., S.A., H.W., D.M.B., and S.M.R. analyzed data; J.C., D.M.B., S.M.R., K.T., L.F., W.W., S.A., and K.M.K. interpreted results of experiments; J.C., K.T., L.F., W.W., S.A., and S.M.R. prepared figures; J.C., K.T., L.F., W.W., K.M.K., and S.M.R. drafted manuscript; J.C., K.T., L.F., W.W., S.A., K.M.K., E.F.L., D.M.B., and S.M.R. edited and revised manuscript; J.C., K.T., L.F., W.W., S.A., H.W., L.T., K.M.K., E.F.L., D.M.B., and S.M.R. approved final version of manuscript.
